# Research Progresses and Challenges of Flexible Zinc Battery

**DOI:** 10.3389/fchem.2022.827563

**Published:** 2022-02-14

**Authors:** Yunfei Xu, Xin Xu, Mei Guo, Guoxin Zhang, Yaqun Wang

**Affiliations:** Department of Energy Storage Technology, Shandong University of Science and Technology, Qingdao, China

**Keywords:** flexible, zinc ion battery, zinc-air battery, mechanical property, flexible substrate, *in situ* growth, inkjet printing, screen printing

## Abstract

Flexible zinc batteries have great potential in wearable electronic devices due to their high safety, low cost, and environmental friendliness. In the past few years, a great deal of work on flexible zinc batteries has been reported, with exciting results. Therefore, many solutions have been proposed in electrode design and electrolyte preparation to ensure the desired flexibility without sacrificing the capacity. This paper reviews the recent progress of flexible zinc batteries. We discuss the differences between various anode materials, cathode materials, and electrolytes, introduce the differences of electrode preparation methods of active materials on flexible substrates and their influence on the performance of the battery. Finally, the challenges and future research trends of flexible zinc batteries in capacity and mechanical properties are pointed out.

## Introduction

With the development of miniaturization of electronic chips, it is possible to integrate electric devices, such as implantable medical devices, wearable health monitoring systems, flexible displays, and intelligent clothing, which have attracted the attention of researchers all over the world. (Yu et al., 2017; Liu et al., 2018; Sumboja et al., 2018; Zhang et al., 2018; Dong et al., 2021).As an indispensable part of the flexible electronic equipment, the battery needs to have good cycling and safety performance to meet the requirements. Especially when the flexible battery follows the deformation of the flexible electronic devices, the mechanical and electrochemical properties are required to be higher.

As an electrode material, zinc has the advantages of lower cost, more content in the crust and lower redox equilibrium potential than lithium. Moreover, using environmentally insensitive zinc makes zinc-based batteries manufacture easier and package cheaper than lithium-based batteries. (Fang et al., 2018). At present, conventional zinc-based batteries such as Zn-MnO_2_, Zn-Ni and Zn-Air have already been commercialized, but they are mainly rigid and used in non-flexible electronic devices. Many researches have been done to make these batteries flexible. In addition, Zinc ion batteries (ZIBs) because of its high energy density, low cost, environmental friendliness, safety and other advantages are gradually coming into people’s horizons, have good prospects in portable devices. ZIB, as a promising alternative to lithium ion battery, has attracted widespread attention. (Tan et al., 2017; Li et al., 2019a; Yu et al., 2019; Zhang et al., 2020a).

At present, great progresses have been made in electrode materials selection, flexible electrode preparation and electrolyte design of flexible zinc ion batteries. (Li et al., 2019a). In this review, the latest research progresses in electrode materials, electrolytes, and adhesion methods of active material are reviewed. Lastly, some concluding remarks were prospected to outline the challenges and future research trends for flexible zinc batteries.

## Common Materials for Flexible Zinc Batteries

### Anode Materials

Zinc metal anodes are of particular interest to the flexible battery market due to their low material cost, high theoretical capacity (820 mA h/g, 5,854 mA h/cm^3^), (Kaveevivitchai and Manthiram, 2016), good rechargeability and safe chemistry. The most common anode in flexible zinc ion battery is zinc foil, because of its good mechanical properties. However, the stiffness of heavy metal zinc foil anodes reduces the energy density, making it impossible to apply to industrial flexible, wearable energy storage systems. (Pan et al., 2016). In addition, the main challenges faced by zinc metal anodes are the mechanical stability of the electrode during long-term deformation and uncontrollable dendrite growth during cycling, resulting in poor cycle performance and coulomb efficiency of the battery, which severely limits the service life of flexible zinc ion batteries and hinders their practical application. (Lao-atiman et al., 2017; Han et al., 2018a; Li et al., 2018a; Ma et al., 2018; Tang et al., 2019; Zeng et al., 2019; Li et al., 2020a; Nguyen et al., 2020). In order to solve these problems of Zn anode, there are several strategies, including:

a) Method for constructing composite zinc anode.

**FIGURE 1:**
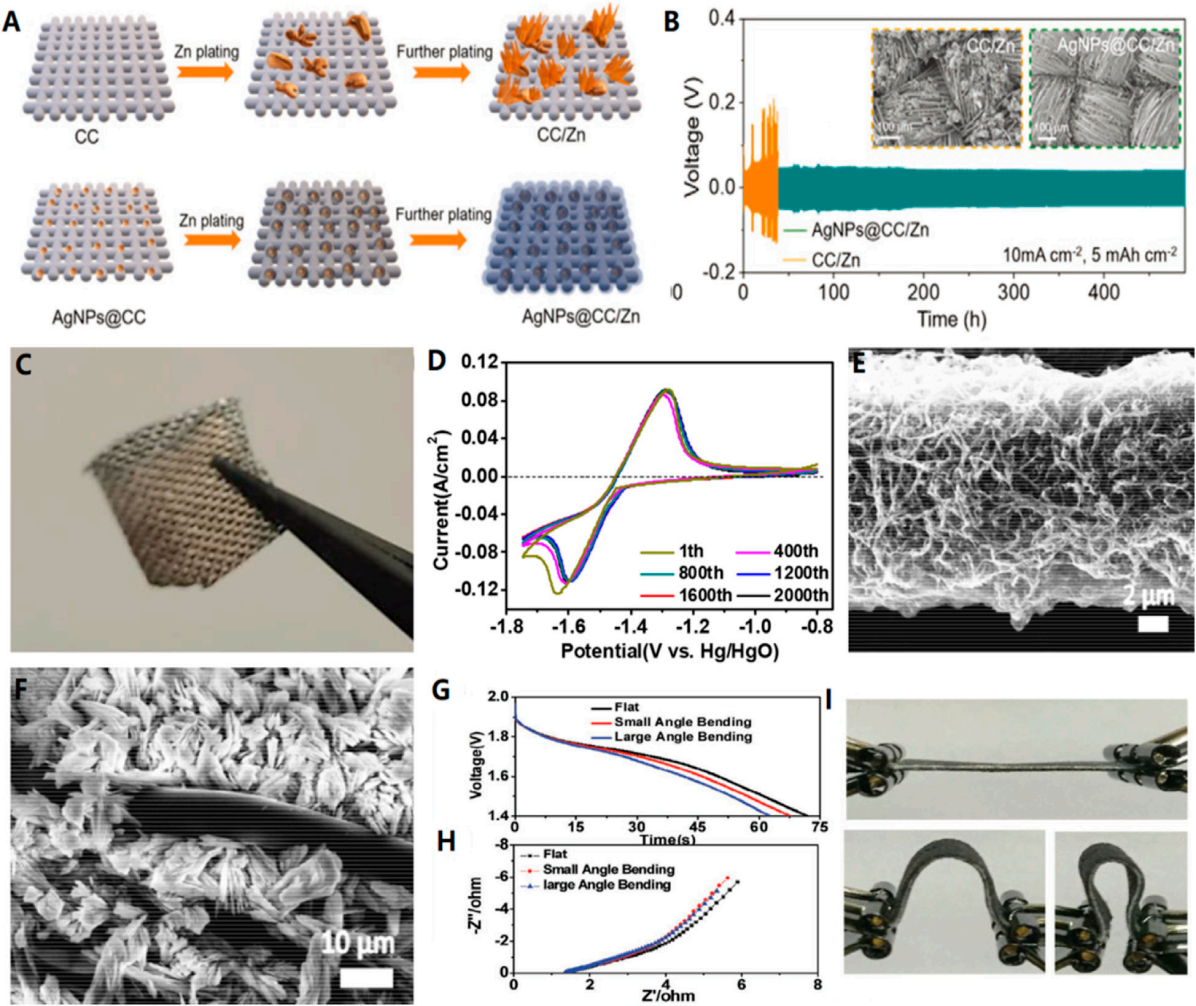
**(A)** Schematic diagram of Zn deposition on bare CC and AgNPs@CC scaffolds. **(B)** The capacity is 5 mA h/cm^2^ at 10 mA h/cm^2^ current density. Inset: SEM images of CC/Zn and AgNPs@CC/Zn electrodes after circulation. **(C)** Flexible AgNPs@CC/Zn electrode under bending.Copyright 2021 Wiley-VCH GmbH. **(D)** CV curves of the CC-CF@120ZnO at different cycles. SEM images of **(E)** CC-CF@ZnO and **(F)** CC-ZnO after cycling test, showing the importance of CFs for preventing the Zn dendrite growth. **(G–I)** Discharge curves and EIS results of solid-state batteries under different bending conditions. Copyright 2016 WILEY-VCH Verlag GmbH & Co. KGaA, Weinheim.

Chen and his colleagues proposed a heterogeneous metal seed-mediated strategy. (Chen et al., 2021a). The basic idea is through the inkjet printing on the main chain of the three dimensional conductive printing silver nanoparticles, in order to induce the homogeneous nucleation of zinc, and avoid initial electroplating phase of the dendrite growth. Therefore, AgNPs@CC/Zn anode has excellent performance at a high current density of 10 mA/cm^2^, with cycle performance exceeding 480 h. (Figures 1A,B)

b) Deposition of zinc materials on a highly conductive substrate.

Zinc oxide nanoparticles are deposited on a three-dimensional layered carbon cloth-carbon nanofiber (CC-CF) substrate as an anode (CC-CF@ZnO). (Figures 1D,E) The device exhibits excellent stability, maintaining up to 91.45% initial capacity after 1,000 cycles and 72.90% initial capacity after 2,400 cycles. The significant increase in cyclic capacity is due to the uniform deposition of nano particles on the three-dimensional highly conductive nano-carbon fibers, which alleviates the shape change during the electrochemical reaction. In particular, it avoids the uneven distribution of current and zinc deposition, thus preventing the formation of zinc dendrite. (Liu et al., 2016).

Pang (Guo et al., 2021) prepared zinc nanosheets/CC (Zn/CC) by electrodeposition, and Zn nanosheets were uniformly deposited on the surface of CC fiber. Form a three-dimensional network interconnected with recent nano piece, insert for the ion-extraction process provides a rich active site. Compared with the same amount of conventional metal zinc anode foil, nanostructures zinc and CC skeleton integration enhances the flexibility of the anode, the formation of the surface of the more active, which helps the development of high performance flexible device.

c) Coating inert material on zinc anode.

For example, (Alfaruqi et al., 2018), constructed a Sn-doped NaTi_2_(PO_4_)_3_ (NTP/Sn) protective layer on the surface of zinc anode (Zn@NTP/Sn) to improve the cyclic stability of zinc anode. Using Sn doping NaTi_2_ (PO_4_) _3_ through the structure design and high performance of zinc anode interface features, is helpful to accelerate the transfer of Zn^2+^, increase the interface performance. At the current density of 0.4 mA/cm^2^, the average voltage lag of Zn@NTP/Sn symmetric cells is only 17.4 mV, which is much lower than that of exposed Zn symmetric cells (75.8 mV) and Zn@NTP symmetric cells (43.7 mV). In addition, it has been reported that the zinc electrode surface was coated with Al_2_O_3_ coating (Lee et al., 2013) and Bi alloying (Jo et al., 2017) to improve HER overpotential and reduce self-discharge caused by hydrogen release, thus improving the cycle performance of the battery.

d) Use appropriate electrolytes.

For example, ionic liquid based zinc salt electrolyte is an effective way to solve the hydrogen evolution reaction (HER) and zinc dendrite growth of zinc ion batteries. (Ma et al., 2020) developed polyvinylidene fluoride hexafluoropropylene (PVDF-HFP)+5% poly (ethylene oxide) (PEO)+ILZE electrolyte (1-ethyl-3-methylimidazolium tetrafluoroboric acid ([EMIM]BF_4_) ionic liquid) using 2 M zinc tetrafluoroborate (Zn(BF_4_)_2_) as carrier. The final product is represented as PHP-ILZE. The electrolyte developed can achieve hydrogen-free, dendrite-free galvanizing/stripping in 1,500 h (3,000 cycles) at 2 mA/cm^2^ with a Coulomb efficiency of nearly 100%. At the same time, oxygen-induced corrosion and passivation were effectively inhibited. This is the first demonstration of an all-solid-state zinc-ion battery based on a newly developed electrolyte, which simultaneously solves the problems of deep hydrogen evolution and dendrite growth of conventional zinc-ion batteries.

Li and his colleagues have developed a polyethylene glycol 600 (PEG 600) and polysorbate 20 (Tween 20) compound additive, as the organic inhibitors in alkaline electrolyte, mainly by inhibiting the hydrogen evolution reaction to a certain extent, inhibit the corrosion of zinc. Because Tween 20 is more polar than PEG 600, it absorbs zinc better. Because of Tween 20 highly branched structure, zinc cannot be Tween 20 complete coverage, and linear polyethylene glycol (peg) can be adsorbed on the rest of the active site, thus two kind of corrosion inhibitor for zinc corrosion synergy effect, the corrosion of the composite inhibitor is better than single corrosion inhibitor. (Liang et al., 2011).

The combination of these methods enables advanced flexible zinc anodes to be better designed with less dendrite growth, higher corrosion and passivation resistance, and less hydrogen evolution, suitable for quasi-solid zinc-based aqueous batteries.

### Cathode Materials

The cathode materials commonly used in flexible zinc ion battery include various transition metal compounds, such as manganese (Qiu et al., 2017; Huang et al., 2019; Zu et al., 2019; Zhang et al., 2020b; Zhang et al., 2020c), cobalt, nickel (Liu et al., 2016; Wan et al., 2018) or molybdenum-based oxides/sulfides, Prussian blue analogues and conducting polymers (Eftekhari et al., 2017; Yao et al., 2020; Cong et al., 2021). (Table 1) Here, we will focus on the last 5 years of research.

**TABLE 1:** Comparison of electrochemical properties of different cathode materials.

Cathode materials	Electrolyte	Capacity	Cycling stability	Power density	Energy density	References
CC-CF@NiO	PVA + ZnO + KOH	0.39 mAh cm^−2^ at 5 mA cm^−2^	91.45% after 1,000 cycles	57.5 W kg^−1^	19.7 Wh kg^−1^	Liu et al. (2016)
N-CC@MnO_2_	(PVA)+LiCl + ZnCl_2_+MnSO_4_	353 mA h g^−1^ at 0.5 A g^−1^	93.6% after 1,000 cycles	7.9 kW kg^−1^	440 W h kg^−1^	Zu et al. (2019)
Ag_2_O	KOH + LiOH + polyacrylic acid	3.78 mA h cm^−2^				Eftekhari et al. (2017)
AgO	KOH + PVA + Ca(OH)_2_	>54 mAh cm^−2^				Cong et al. (2021)
CC-PANI-FeCN	ZnSO_4_+PVA	238 mAh/g at 0.2A/g	71% at 1,000 cycles			Zhang et al. (2015)
ZnHCF@MnO_2_	ZnSO_4_+PVA	89 mA h/g at 100 mA/g	∼71% at 500 cycles			Wang et al. (2017)
CoFe(CN)6	PAM + poly (ethylene oxide)_53_-poly (propylene oxide)_34_-poly (ethylene oxide)_53_ (F77) +Zn(OTf)_2_	173.4 mAh g^−1^ at 0.3A/g	93.4% after 2000 cycles			Javed et al. (2020)
AgNPs@CC	Zn(CF_3_SO_3_)_2_	255mAh/g at 5.0 mA/g	184 mAh/g at 1,200 cycles			Chen et al. (2021a)
P-MnO_2−x_@VMG	PVA + ZnCl_2_+MnSO_4_	284.2 mA h/g at 0.5A/g	＞87% at 500 cycles		369.5 Wh kg^−1^	Chamoun et al. (2018)
t-CNTs-PA-PE	ZnSO_4_+PAM	238 mA h/g at 0.2 A/g	∼100% at 1,500 cycles			Jia et al. (2015)
CC-MOF derived Ag nanowires	KOH + PVA	1.605 mA h/cm^2^ at	CC-MOF derived Ag nanowires	2.8 mW/cm^2^	1.87 mWh/cm^2^	Tehrani et al. (2015)
MnO_2_	ZnSO_4_+ MnSO_4_	19.3 mAh/cm3 (corresponding to 393 mA h/g) at7.5 mA/cm^3^	83.9% at 1,300 cycles		38.1 mW h cm^−3^	Wang et al. (2018b)
PANI/mCC	PVA + Zn(CF_3_SO_3_)_2_	156.4 mAh g^−1^	80% after 1,000 cycles	9742 W kg^−1^	185.7 Wh kg^−1^	Guo et al. (2021)
CNT/MnO_2_-PPy	ZnSO_4_+ MnSO_4_	222.7 mAh/g at 0.3A/g	87.4% after 1,000 times		341.6 Wh kg^−1^	Yin et al. (2021)
V_2_O_5_-Ti	Zn（CF_3_SO_3_）_2_ + PVA	377.5 mAh g^−1^ at 4A g^−1^	68.21% after 500 cycles	6.4 kW kg^−1^	622 Wh kg^−1^	Song et al. (2016)
binder-free V_2_O_5_ nanorods	PAM + ZnSO_4_	362.01 mAh g^−1^ at 0.13 A g^−1^	81.9% after 600 cycles	33.4 mW cm −3	10.5 mWh-cm^−3^	Lin et al. (2020)

#### Manganese Based Materials

**FIGURE 2:**
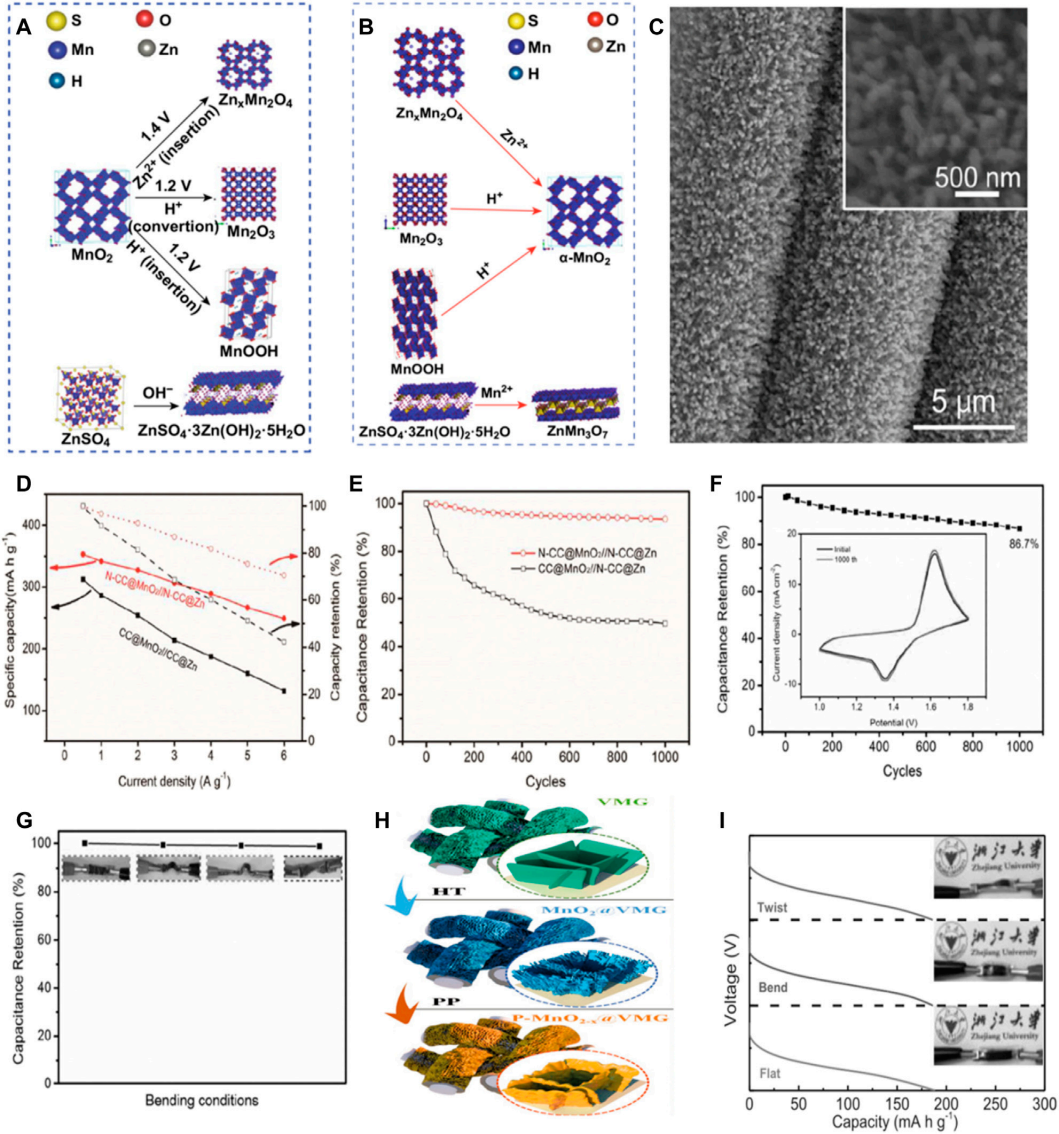
Phase evolution of cathode during the first discharge process **(A)** and charge process **(B)**. Copyright 2019 Springer. **(C)**SEM images of the N-CC@MnO_2_; inset is the corresponding magnifed SEM image. **(D)** N-CC@MnO_2_//N-CC@Zn and CC@MnO_2_//N-CC@Zn the specific capacity and capacity retention rate of the battery as a function of current density **(E)** Cycling performance of the N-CC@MnO_2_//N-CC@Zn and CC@MnO_2_//N-CC@Zn batteries collected at 1 A/g for 1,000 cycles. **(F)** Capacity retention rate of the battery after 1,000 cycles at the current density of 1 A/g N-CC@MnO_2_//N-CC@Zn. The CV curves of the first and 1000^th^ cycles are illustrated. **(G)** At different bending conditions N-CC@MnO_2_//N-CC@Zn battery capacity retention rate.Copyright 2017 Journal of Materials Chemistry A. **(H)** For the synthesis of MnO_2-x_ @ VMG shell/core array. (HT, hydrothermal method; PP, phosphorization process). **(I)** Discharge curves of the device under flat, bending and twisting conditions at 3 A/g current density. Copyright 2020 WILEY-VCH Verlag GmbH & Co. KGaA, Weinheim.

Manganese based compounds have been widely used as cathode materials for ZIB due to their advantages of abundant resources, low cost and non-toxicity. (Pan et al., 2016; Zhang et al., 2016). MnO_2_ is considered as the most promising cathode material among many manganese based compounds. MnO_2_ is a material with a polymorphic crystal shape. Under different preparation conditions, the crystal shape, morphology, particle size, porosity, specific surface area are different, and then show different capacity and charge-discharge characteristics. (Xu et al., 2012; Alfaruqi et al., 2018). Huang and his colleagues have provided new insights into the energy storage mechanism of Waterborne zinc-manganese battery with the participation of Mn^2+^. (Huang et al., 2019). In the first discharge process, the combination of Zn^2+^ and H^+^ insert promoted the MnO_2_ to Zn_x_MnO_4,_ MnOOH and Mn_2_O_3_ transformation, at the same time, raised the pH of the electrolyte, formation of ZnSO_4_⋅3Zn(OH)_2_⋅5H_2_O (noted as “BZSP”). Later in the charging process, Zn_x_MnO_4_, MnOOH and Mn_2_O_3_ by extracting Zn^2+^ and H^+^ reduction for alpha MnO_2_, BZSP and Mn^2+^ reacts ZnMn_3_O_7_ ⋅3H_2_O. Besides the electrochemical reaction, Zn^2+^ can also be in alpha MnO_2_, ZnxMnO_4_ and ZnMn_3_O_7_⋅3H_2_O in the reversible insert and extract, under the participation of Mn^2 +^, BZSP, Zn_2_Mn_3_O_8_ and ZnMn_2_O_4_ reciprocal transformation (Figure 2B).

On the other hand, the slow kinetics and rapid capacity decay caused by the strong electrostatic repulsion between the divalent zinc ions and the manganese based host crystal structure is one of the important challenges for the practical application of zinc ion batteries. Li’s team proposed *in-situ* growth of manganese dioxide nanorods arrays on three-dimensional porous surface nitrogen-doped carbon cloth (N-CC) (Figure 2C). It is not only better than the other nanostructures has higher specific surface area, more active site and better permeability, and can maintain the stability of the structure and electrochemical significantly improve the conductivity at the same time. In addition, it allows a strong covalent coupling based on the nanoscale active material and N-CC, leading to a fast electron transfer from the active material to the collector. Thus, Under the current density of 0.5 g/A, flexible-CC@ MnO_2_/N-CC@Zn battery can produce a high capacity of 353 mA h/g (Figures 2D,E). In addition, the CV curve of our assembled state N -CC@MnO_2_//N-CC@Zn battery hardly changes after 1,000 cycles, which also proves that the cell has good cycle stability. Further, the prepared devices can be bent and twisted in different states without affecting their electrochemical performance (Figures 2F,G). (Qiu et al., 2017)

In addition, many researchers believe that the oxygen vacancy generation and ion intercalation are another effective method to improve the electrochemical performance of Zn/MnO_2_ battery. There are two main reasons: On the one hand, as shallow bodies, oxygen vacancies can fundamentally improve the electrical conductivity of manganese-based materials, influence the embedding and de-embedding of metal ions in material layers, reduce the stress and electrostatic repulsion between adjacent layers, directly overcome migration and diffusion barriers, and promote the diffusion and charge transfer of ions during the embedding of metal ions; on the other hand, the electrode material with oxygen vacancy can also generate more electrochemically active sites, increase the surface energy of the system, promote the electrochemical phase transition, and thus possess better charge storage capacity. (Zu et al., 2019; Zhang et al., 2020b).

Zhang’s team reported a simple phosphorylation process that introduces oxygen vacancies into phosphate ion intercalated manganese dioxide/vertical multilayer graphene (VMG) arrays to form complete P-MnO_2-x_@VMG cathodes (Figure 2H). By phosphorylation, the oxygen vacancy and phosphorus ion intercalation are realized simultaneously, which improves the electrical conductivity of MnO_2_ and expands its layer spacing to accelerate ion transfer. In addition, the flexible VMG conductive network provides good peripheral charge transfer and imparts good mechanical strength to the cathode. Thanks to these advantages, at the current density of 0.5 A/g, P-MnO_2-x_@VMG cathodes shows a high capacity of 302.8 mA h/g in aqueous electrolytes, and the capacity retention rate is more than 90% after 1,000 cycles at the current density of 2 A/g, which proves its long-term cycling stability. In addition, by comparing the 3 A/g discharge curves of the battery under flat, bent and twisted conditions, it can be seen that the difference between the three curves is negligible, which proves that the battery has great potential in flexible wearable electronic devices. (Figure 2I). (Zhang et al., 2020c) Mai et al. reported the generation of oxygen vacancies in tunnel α -MnO_2_ via surface gradient Ti doping using defect engineering for long-life Zn-MnO_2_ batteries. (Lian et al., 2019). Interestingly, the introduction of surface gradient Ti doping leads to the contraction of the interlayer, but at the same time, the reduction of Mn valence state leads to the formation of oxygen vacancies compensated by electrons. In addition, Ti substitution and the resulting oxygen vacancy open the [MnO_6_] octahedral wall, resulting in an unbalanced charge distribution and local electric fields in the crystal structure, accelerating ion/electron migration rates. Therefore, the diffusion coefficients of Zn^2+^ and H^+^ in Ti-MnO_2_ nanowires are improved. Therefore, Ti-MnO_2_ nanowires show improved H^+^ and Zn^2+^ storage capacities in Zn/MnO_2_ batteries, and achieve excellent high rate capacity and ultra-long cycle stability.

Also, in ZnSO_4_ electrolytes widely studied, MnO_2_ usually suffers volume loss due to Mn^3+^ disambiguation dissolving Mn^2+ 39^. Mn^2+^ is added to the electrolyte to inhibit the disproportionation of Mn^3+^, which can effectively inhibit the dissolution of manganese base materials and improve the stability of cathode materials (Pan et al., 2016; Chamoun et al., 2018), but the mechanism is still unclear. For example, in a Zn-MnO_2_ cell, dissolution of α -MnO_2_ is alleviated when MnSO_4_ is added to a ZnSO_4_ electrolyte because it can alter the dissolution balance of Mn^2+^ from the α-MnO_2_ electrode. (Lian et al., 2019). Zhang and his colleagues reported MnO_2_ based anode and cathode, zinc added Mn(CF_3_SO_3_)_2_ additives of Zn(CF_3_SO_3_)_2_ electrolytic Zn-MnO_2_ water of high performance rechargeable batteries. It is found that Mn(CF_3_SO_3_)_2_ can inhibit the dissolution of Mn^2+^ and form uniform porous MnO_x_ nanosheets on the cathode surface, which helps to maintain the integrity of the electrode. It is important to note that beta MnO_2_ has the high irreversible capacity, stable high rate capability and cycle performance. (Zhang et al., 2017). In addition, coating can also be used to alleviate the dissolution of mangan-based materials. For example, Yang et al. prepared an independent flexible film (Designated as CNT/MnO_2_-PPY) composed of carbon nanotubes and polypyrrole coated manganese dioxide nanowire through *in-situ* reactive self-assembly and vacuum filtration. The Polypyrrole (PPy) coating improves the conductivity of MnO_2_ NWs, alleviates the dissolution of cathode materials and provides a strong buffer to accommodate large volume changes during repeated cycles. (Zhang et al., 2020d).

### Silver-Based Materials

**FIGURE 3:**
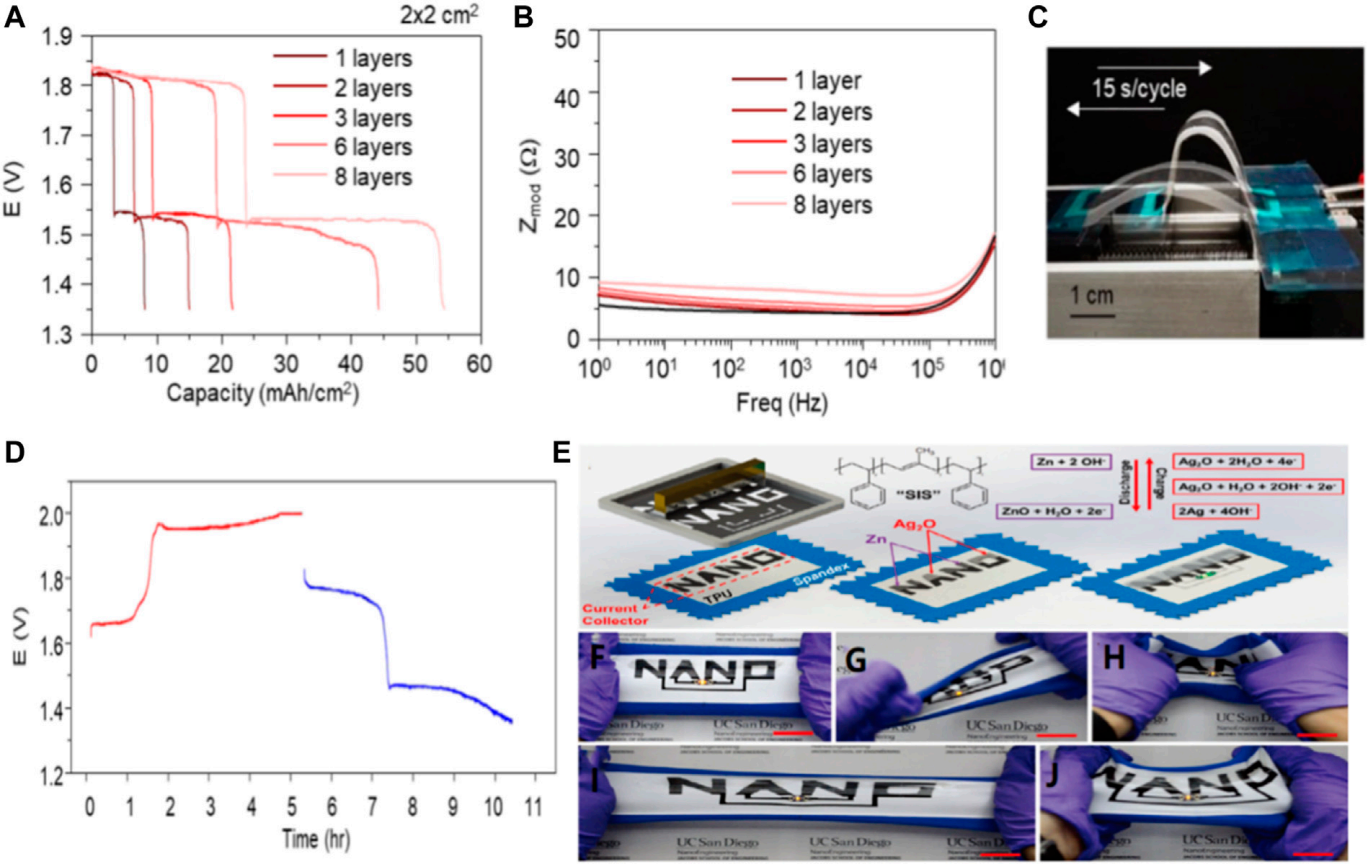
**(A)** Electrochemical performance of AgO-Zn batteries as galvanic cells. The active material load is the capacity of the 2 × 2 cm^2^ battery with 1-8 layers. **(B)** Bode plot reflecting the corresponding impedance of the 2 × 2 cm^2^ cells with different areal loading. **(C)** Diagram of a cell controlled by a linear stage at a speed of 15 s/cycle under repeated 180° bending cycles, and **(D)** corresponding volt-time curves for charging (red) and discharging (blue) of the cell during approximately ∼2,500 repeated bending cycles. Copyright 2020 ResearchGate. **(E)** Screen printing steps for Zn-Ag_2_O cells on stretchable fabric using SIS adhesive. Illustration: REDOX charging-discharging reaction. Photographs of the sealed cell during **(F)** horizontal stretching, **(G)** distortion, **(H)** indentation strain, **(I)** 100% stretching and **(J)** biaxial stretching. Copyright 2016 Advanced Energy Materials.

Zinc-silver batteries have the following advantages: high specific energy (theoretical specific capacity: 432 mAh/g), high discharge efficiency, moderate charging efficiency, smooth discharge voltage, small self-discharge rate, long dry storage life and good mechanical properties. (Kumar et al., 2019; Tan et al., 2018). At the same time, zinc-silver batteries also have obvious shortcomings, which are as follows: very high cost, short life, poor low-temperature performance and not resistant to overcharging. The silver oxide button battery has widely used in electronic watches, calculators, small instruments and other micro electrical appliances as power supply because of their above-mentioned advantages. In recent years, a lot of research has been done to make these batteries flexible. In addition, Wang’s team has developed a printable, polymer-based zinc-silver oxide (AgO-Zn) battery with flexibility, rechargeability, high area capacity and low impedance. The redox reactiondepends on the zinc ions (Zn^2+^) and silver ions dissolved in alkaline electrolyte (Ag) and the supersaturated induced precipitation. In addition, due to the high oxidation state, make full use of line printing batteries can realize >54 mA h/cm^2^ area of high capacity (Figure 3A) while maintaining a low internal resistance (Figure 3B). The battery can still charge and discharge normally without multiple deformations affecting its rechargeability (Figures 3C,D). Overall, printed thin film AgO-Zn thin film batteries combine superior electrochemical and mechanical properties and prove to be well suited for reliably and sustainably powering a wide variety of wearable and flexible electronic devices. In addition, Meng and his colleagues used polystyrene block polyisoprene block polystyrene (SIS) as a hyperelastic adhesive for printing inks to print a Zn-Ag_2_O battery with high ductility and a reversible capacity density of 2.5 mA h/cm^2^ even after repeated 100% stretching.Two stretchable battery with “NANO” design printed directly on the seal on the spandex thermoplastic polyurethane (PU) the top of the head (Figure 3E). On the “NANO” current collector, electrodes are printed separately to form two “NA” and “NO” batteries designed to be connected in series to power the 3V wearable LED (Figure 3F). Regardless of the serious torsional strain (Figure 3G), indentation (Figure 3H), 100% uniaxial tensile (Figure 3I) and biaxial stretching, stretching “NANO” to the battery can keep constant LED brightness (Figure 3J). The battery energy provided so far reported the highest reversible capacity of the inherent scalability battery size and discharge current density. New stress wear-resisting printing inks for the wide application of flexible paved the way to the further development of the electronic products. The new printed battery based on SIS can withstand severe bending caused by the wearer’s movement. (Kumar et al., 2016).

### Conductive Polymers

**FIGURE 4:**
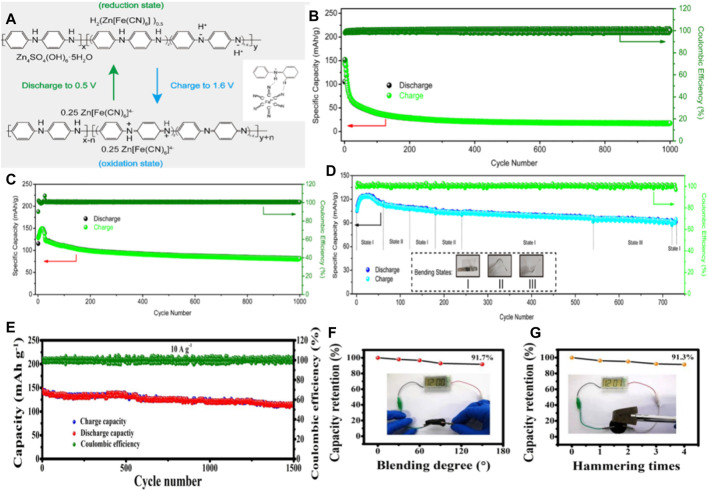
**(A)** REDOX mechanism of CC-PANI-FeCN. Illustration[Fe(CN)_6_]^4−^ Schematic diagram of interaction with PANI. Cyclic properties of CC-PANI **(B)** and CC-PANI-FeCN **(C)** at current density 5 A/g. **(D)** The optical images ofquasi-solid-stateflexible batteries in different bending states. Copyright 2020 Chemical Engineering Journal. **(E)** Cycling performance of t-CNT-PA-PE cathode in ZIBs at current density of 10 A/g. Capacity retention of solid ZIBs under different destructive tests, including **(F)** bending test and **(G)** hammer testCopyright 2019 ACS Appl Mater Interfaces.

Compared with metal oxides, conductive polymers exhibit higher conductivity due to their long π -electron conjugated system (Shi et al., 2018), and also has the advantages of low cost, easy synthesis, and excellent mechanical properties (Cong et al., 2021), are excellent cathode materials for Zn batteries, and show promising energy storage behavior. Therefore, conductive polymers have attracted wide attention in the field of electric energy storage, especially in wearable electronic devices. Polyaniline (PANI) is one of the most common type of conductive polymers. Polyaniline is easy to synthesize and has high electrical conductivity and electrochemical activity. (Eftekhari et al., 2017). It as a stream of zinc ion battery cathode material has been widely studied. However, it suffers from rapid deactivation and consequent performance deterioration due to spontaneous deprotonation during charging/discharging, and has the disadvantage of poor cycling stability in high pH solutions, which limits its further application in polyaniline zinc batteries. Therefore, Yang’s group reported a strategy to introduce [Fe (CN)_6_]^4-^, in which the nitrogen atoms on the polyaniline chain may also interact with [Fe(CN)_6_]^3-^/[Fe(CN)_6_]^4-^ to affect the electrochemical stability of polyaniline. Considering these factors, [Fe(CN)_6_]^3-^/[Fe(CN)_6_]^4-^ and polyaniline chain between the REDOX reaction and hydrogen bonding may be beneficial to the electrochemical properties of polyaniline, which can greatly improve the cycle stability of the polyaniline electrode, at the same time maintain its initial high specific capacity (Figure 4A). After 1,000 times charging and discharging cycle, CC–PANI–FeCN capacity retention rate of about 71%, and the capacity of the CC–PANI retention rate is only 17% (Figures 4B,C). Assembly of quasi solid flexible zinc ion battery under the condition of different bending the specific capacity and coulomb efficiency is close to 100%, suggest that the potential of as flexible energy storage device (Figure 4D). (Yao et al., 2020)

In addition, Huang’s team reported a method to improve the electrochemical reactivity and stability of the polyaniline cathode by constructing a pile-electron conjugate system between PEDOT: PSS on polyaniline and carbon nanotubes. The cathode can provide a high capacity of 238 mA h/g at a current density of 0.2 A/g, with good rate performance and good cycle stability (Figure 4E). Zinc ion batteries based on post-treated CNT PANI PEDOT: PSS (t-CNTs-PA-PE)showed good cycle stability and almost 100% cycle efficiency in 1,500 cycles. Moreover, Solid ZIBs in the bending and hammering process, the capacity loss is not obvious, keep in more than 90% of the original capacity (Figures 4F,G). This work demonstrates that conductive polymer cathodes can be used in high-performance ZIBs to meet the needs of flexible electronics. (Liu et al., 2019a).

#### Prussian Blue Materials

PBA with the formula of A_x_M_y_ [B(CN)_6_]_z_mH_2_O (x, y, z, m = sto-ichiometric numbers; A, B = alkaline metal; M = Zn^2+^, Ni^2+^, Cu^2+^, etc.) is constructed by a 3D network of zeolitic struc-ture. (Xu et al., 2017). It has open skeleton structure, sufficient REDOX active center and relatively strong structural stability (Jia et al., 2015; Zhang et al., 2015; Song et al., 2018). The open framework endows PBA to be promising candidate for Zn^2+^ storage. However, Prussian blue analogues have a low capacity (less than 100 mAh/g) because the active sites are underutilized and in most cases are single-atom REDOX (Ma et al., 2019a). It is still challenging to further improve the specific capacity and cycling stability of PBA cathodes.

**FIGURE 5:**
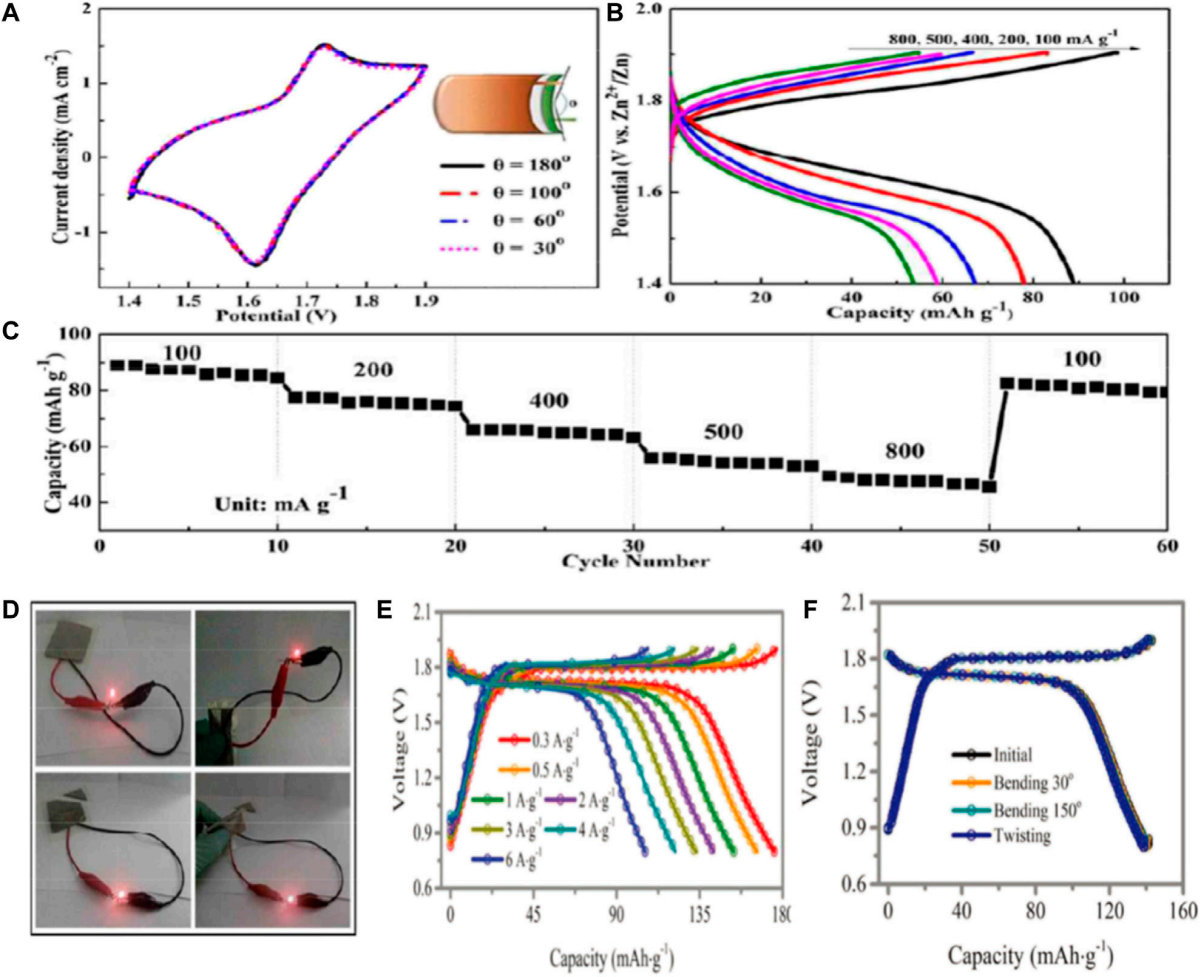
Performance of flexible solid-state rechargeable zinc ion battery with ZnHCF@MnO_2_ nickel foil as cathode **(A)** CV curves under different bending conditions at 1 mV/s; **(B)** Charge-discharge curves under different current densities; **(C)** Multiplier performance of flexible cells; **(D)** Photographs of the flexible zinc batteries powering the LED bulbs in their original and cut state respectively Copyright 2017 J. Mater. Chem. A. Electrochemical performance of Zn/CoFe(CN)_6_ cells with 4 m Zn(OTf)_2_ electrolyte **(E)** constant current charge-discharge curves under different current densities; **(F)** Solid-state cable battery: constant current charge-discharge curve and torsional deformation of battery at different bending angles of 60° and 150° when the fixed device length is 10 cm and bending radius is 1 cm.

Lu and his colleagues prepared manganese oxide coated zinc ferrite (ZnHCF) nanocubes (ZnHCF@MnO_2_) by *in situ* coprecipidation method (Lu et al., 2017). The composite has a unique structure that has a synergistic effect by combining the capacitive and intercalated properties of the two components with the REDOX reaction, thus regulating the storage of Zn ions. Therefore, ZnHCF nanocubes encapsulated by MnO_2_ nanocubes have a high capacity of Zn ion storage and discharge, and their working voltage can reach ∼1.7V. In order to demonstrate the practical application of zinc ion battery in the field of flexible wearable electronics, a flexible quasi-solid-state battery was prepared by coupling ZnHCF@MnO_2_ with Zn thin foil in ZnSO_4_/PVA gel electrolyte. At different bending angles, the battery’s current changes are negligible, showing excellent flexibility (Figure 5A). In addition, the discharge capacities of the flexible device at 100, 200, 400, 500 and 800 mA/g are 89, 78, 67, 58, and 53 mAh/g, respectively (Figure 5B). In addition to higher discharge capacity, quasi-solid state batteries also have higher rate capacity (Figure 5C). When the current density increases from 100 mA/g to 800 mA/g, the discharge capacity can still exceed 49 mA h/g, and the capacity retention rate is 55%. At the same time, flexible battery can be stable cycle more than 500 times, can maintain about 71 percent of the capacity. Even flexible battery folding can power LED bulbs (drive voltage is 1.8V). (Figure 5D).

Zhi and his colleagues incorporated Co(II)/Co(III) and Fe(II)/Fe(III) REDOX reactions into cobalt hexocyanate (CoFe(CN)_6_), which is a breakthrough to realize the combination of high capacity and high voltage water-bearing zinc ion batteries. (Ma et al., 2019a). The Zn/CoFe(CN)_6_ battery utilizes two pairs of Co(II)/Co(III) and Fe(II)/Fe(III) REDOX reactions to provide a high operating voltage platform of 1.75 V and a high capacity of 173.4 mA h/g at 0.3 A/g. The 3D open structure of the battery provides A sufficiently high discharge capacity of 109.5 mA h/g, even at an extremely fast charge/discharge rate of 6 A/g. (Figure 5E). In addition, the Zn/CoFe(CN)_6_ battery achieved excellent cycle performance of 2,200 times and coulomb efficiency of nearly 100%. Furthermore, sol-gel of hydrogel electrolyte has been developed to prepare high-performance flexible cable batteries. The battery has excellent electrochemical performance, uniform torsional deformation and excellent mechanical properties at bending degrees of 60° and 150°. (Figure 5F). This strategy enables the active material to fully contact the electrolyte, thus improving the electrochemical performance (capacity increase of ≈18.73%) and mechanical stability of the solid-state device.

### Vanadium Base Material

Vanadium cathode because of its excellent REDOX chemistry, can promote the open of insert/take off layer response the main structure and high specific capacity and aroused people’s great interest. (Wang et al., 2017; Guo et al., 2018). Among various vanadium based oxides, V_2_O_5_ has good potential as ZB cathode material due to its high theoretical capacity (589 mA h/g) based on double electron transfer. The main challenge facing V_2_O_5_ cathode is that the V_2_O_5_ cathode synthesized by traditional methods has insufficient active sites, low conductivity, making its ultra-fast charging performance and high rate performance far below expectations.

**FIGURE 6:**
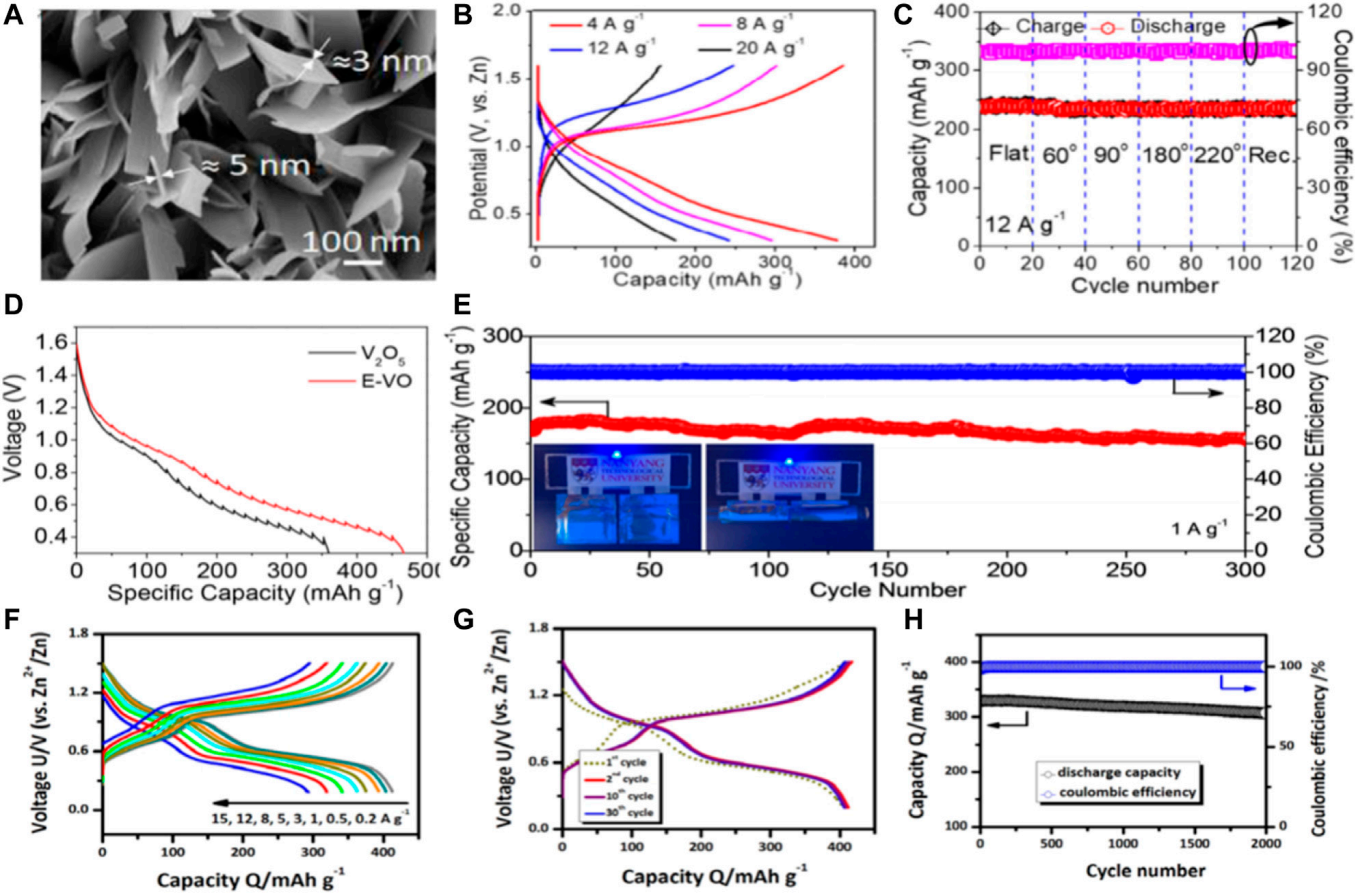
**(A)** High power FESEM images of V_2_O_5_ nanosheets grown directly on titanium substrates, **(B)** Charge-discharge curves of at various current densities in potential window of 0.3–1.6 V, **(C)** Charge-discharge profiles during different bending states at constant current density of 12 A/g. Copyright *2020 Elsevier Ltd.* **(D)** Discharge GITT curves at a current density of 0.05 A/g,**(E)** Cycling stability and Coulombic efficiency at 1 A/g. Inset in **(E)** shows the blue LED light up by two flat or bending state flexible quasi-solid-state Zn/E-VO batteries. Copyright 2019 NANOEN 3724. **(F)** Charge/discharge curves of the DVOC sample at different current densities. **(G)** Charge/discharge curves of the DVOC sample during the initial thirty cycles. **(H)** High-rate long-term cycling properties of the DVOC sample at 10 A/g. Copyright 2020 ChemSusChem.

Mai and colleagues designed a novel cathode material by growing two-dimensional V_2_O_5_ nanosheets directly on a flexible titanium (Ti) substrate. (Javed et al., 2020). The resistance of 2D V_2_O_5_ nanosheets is greatly reduced and provides more active sites to facilitate electrochemical reactions in zinc ion batteries. Meanwhile, the improvement of the electrochemical performance of V_2_O_5_-Ti cathode is due to the unique structure of vertically arranged ultra-thin nanosheets (Figure 6A) which ensures that Zn^2+^ has a larger embedding space and a shorter diffusion length, thus improving the electrochemical performance and providing ultra-fast charging performance. An ultra-fast and flexible quasi - solid F-V_2_O_5_-Ti//Zn battery was developed. The capacity, power density and energy density parameters are better than those of the previously reported water-based/flexible zinc ion battery. (Figures 6B,C).

Yan and colleagues propose a simple *in situ* method that simultaneously introduces polyvalence to increase the interlayer water content and enlarge the interlayer distance of hydrated V_2_O_5_. (Zhao et al., 2019). These structural adjustment makes the layered expansion V_2_O_5_ 2.2 H_2_O (E-VO) nano piece has faster charge transfer kinetics, Zn^2+^storage space and more than precursor V_2_O_5_ higher structural stability. (Figure 6D). As cathode of water-based ZIB, E-VO has the high irreversible capacity (450 mA h/g at 0.1 A/g), good ability of ratio (222 mA h/g at 10 A/g) and long term stability (72% of the capacity to keep 3,000 cycle, at 5 A/g). (Figure 6E).

In addition, the structure crushing and chemical dissolution during the charge-discharge cycle are the main reasons for the electrochemical instability of vanadium oxide. (Yu et al., 2015; Song et al., 2016). In order to solve these problems, regulating the V^4+^ and the ratio of the V^5+^ to enhance the electrochemical reaction of reversibility, effectively improve the capacity and stability of the vanadium oxide. An oxygen defect modulated unbonded V_2_O_5_ nanorods (PVO@C) for water/quasi-solid zinc ion batteries were constructed. (Liang et al., 2021). The structure of PVO@C electrode is stable due to oxygen deficiency and phosphorus doping, and the diffusion rate and electronic conductivity of Zn^2+^ ions are improved. Aqueous PVO@C//Zn cells have significantly increased capacity (362.01 mA h/g at 0.13 A/g1) and have remarkable long-term durability (86.7% capacity after 5,000 cycles and nearly 100% Coulomb efficiency) compared to the original VO//Zn or PVO//Zn cells. In addition, a stable flexible quasi-solid PVO@C//Zn cell (SS ZIB) was proposed based on flexible PVO@C cathode, elastic Zn anode and PAM gel electrolyte. This SS ZIB device achieves an impressive energy density of 10.5 mWh/cm^3^ at a high power density of 33.4 W/cm^3^, exceeding most solid-state batteries previously recorded. After 600 cycles, it has a capacity retention capacity of 81.9%.

Vanadate based materials have the advantages of various element valence states, large theoretical capacity, stable structure and abundant resources, so they are the most widely studied. (Li et al., 2020b; Liao et al., 2020). However, due to strong electrostatic interaction with divalent zinc ion the vanadate cathode has slow kinetics and poor cyclic stability. Deng and colleagues (Lin et al., 2020) constructed a novel vanadate oxide structure and successfully designed a defection-rich (V_6_O_13_-δ)/C nanovortex to prepare a fibrous flexible DVOC/SWNT@CNTF electrode (SWNT: single-walled carbon nanotube; Carbon nanotube fiber CNTF was prepared). Based on SWNT network and substrate are provided based on CNTF bicontinuous electronic path, promoted the fast dynamics and excellence rate capability (Figures 6F–H).

In addition, the pre-insertion strategy is also an effective method to improve the cycling performance of vanadium-based zinc ion batteries. Linda F. Nazar and colleagues report a vanadium oxide bronze supported by interlaminar Zn^2+^ ions and water (Zn_0.25_V_2_O_5_•nH_2_O) as the positive electrode of a zinc battery. (vKundu et al., 2016). A reversible Zn^2+^ ion (de) intercalation storage process with high speed and more than one Zn^2+^ per formula unit (capacity up to 300 mAh/g). The zinc battery has an energy density of 450 W/L and a capacity retention rate of more than 80 percent in 1,000 cycles. No dendrites form on the zinc electrode. In order to solve the problem, the preintercalated ions can easily lose the combination with the skeleton and deintercalated into the electrolyte, leading to the collapse of the structure again.Xu reported that the cyclic stability of a zinc ion cell can be improved by preintercalation of tetrapalent tin ions into Pyrovanadate Sn_1.5_V_2_O_7_(OH)_2_•3.3H_2_O (denoted as SnVO). (Xu et al., 2021). Compared with Pure vanadium pentoxide, the tetradvalent tin in SnVO can strongly bind to V_2_O_7_^4−^ layer, supporting high mechanical stability during zinc ion intercalation. In addition, the tin oxide tetrahedron in the V_4_O_10_ layer can further expand the size of the cavity between Pyrovanadate V_2_O_7_^4−^, promoting the rapid kinetics of zinc ion diffusion, thus improving the rate performance of ZIB.

### Electrolytes

Electrolyte is another important component of flexible zinc ion battery, which is the “blood” of flexible zinc ion battery and plays an important role in determining its performance in terms of discharge operating time, cycling performance and shelf life. There are two types of electrolytes, one is liquid electrolyte (Hilder et al., 2009), which has a certain disadvantage compared with gel electrolyte because of its fluidity and is prone to side leakage when encapsulating flexible batteries. Next is the solid electrolyte, which contains three main types: hydrogel, ionic conductive inorganic solid and organic polymer. Gel polymer electrolyte is a kind of intermediate state between liquid and solid, which is composed of polymer object and liquid electrolyte. Due to its relatively high ionic conductivity, flexibility and good interfacial contact with electrodes, it has been widely used in flexible zinc ion batteries. In general, the performance of gel electrolytes depends largely on the choice of gelatin and the ratio of each element. The gelatins used to build electrolytes are generally polyvinyl alcohol (PVA), polyethylene oxide (PEO), and polyacrylic acid (PAA) (Li et al., 2019a). Polyvinyl alcohol (PVA) is a commonly used polymer matrix with good water retention capacity, which has the characteristics of high hydrophilicity and good film forming ability, excellent chemical stability, electrochemical inertness, durability, non-toxicity and ease of manufacture. (Li et al., 2019b; Fan et al., 2019).

**FIGURE 7:**
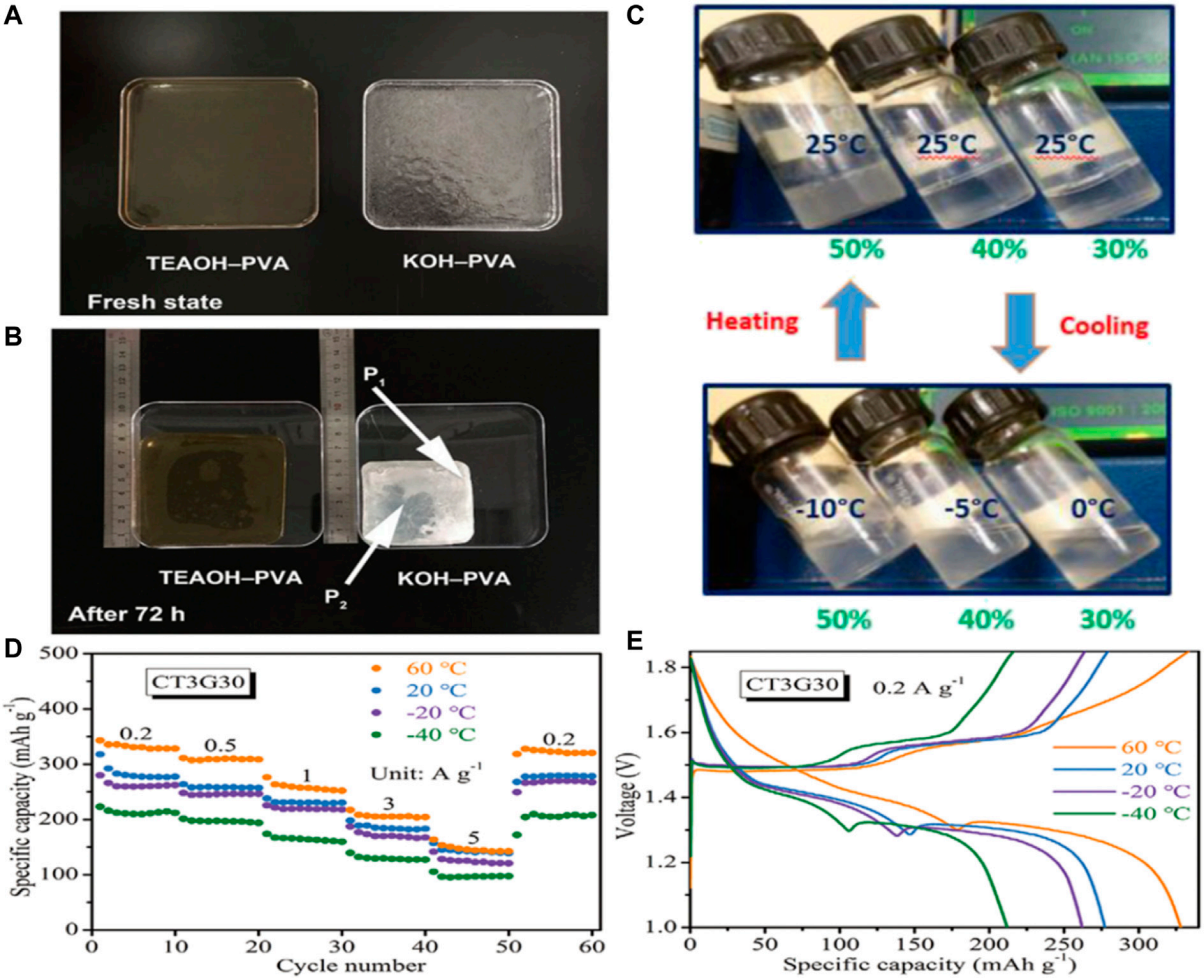
Optical photos of **(A)** freshly prepared electrolytes and **(B)** electrolytes stored in the open ambient environment (RH = 30%, 20°C) for 72 h.Copyright 2019 Nano Energy. **(C)** Photographs of PHEs with polymer concentration ranging from 30% (w/w) to 50% (w/w) at varied temperatures, showing the thermoreversible gelation behavior. Copyright 2017 Angewandte Chemie International Edition. **(D)** Rate performance, **(E)** GCD curves at 0.2 A/g. Copyright 2021 Wiley-VCH GmbH.

As the evaporation of water, the electrolyte becomes more and more concentrated and the performance of battery deteriorates. To reduce water loss of the electrolyte, thereby improving the ionic conductivity of the electrolyte and the cycle life of battery, Zhong’s team has proposed a polymer electrolyte for the body that uses tetraethylammonium hydroxide (TEAOH) as an ionic conductor and polyvinyl alcohol (PVA) as a polymer, with good water retention (Figures 7A,B). The prepared polymer electrolyte maintained a high ionic conductivity of 30 mS/cm after 2 weeks. In addition, with the commonly used KOH-PVA electrolytic liquid ratio, TEAOH - PVA assembly zinc air battery has excellent discharge properties and cycle life, found no significant degradation in 2 weeks. (Li et al., 2019b).

In addition, the internal pores of the gel electrolyte play a key role in the absorption of electrolytes, electrolytes can be retained in the porous gel electrolytes, and polyethylene glycol as a pore-forming agent improves the doping ability and obtain high ionic conductivity. The prepared porous photovoltaic nanocomposite GPE has high ionic conductivity of 57.3 mS/cm, excellent water retention performance and good thermal and mechanical properties under environmental conditions, while the assembled flexible ZAB has excellent cycle stability, discharge performance and power density. (Fan et al., 2019).

In addition to the ionic conductivity and mechanical strength of the hydrogel, certain properties of the polymer electrolyte may have a particular impact on the performance of flexible zinc ion batteries. (Li et al., 2018b). For example, Cui’s team reported a novel strategy using thermally reversible polymer hydrogels as functional electrolytes, i.e., using poly (ethylene oxide)-poly (propylene oxide)-poly (ethylene oxide) (PEO-PPO-PEO), with significant structural polymorphism in polar solvents, to provide intelligent cooling recovery for flexible zinc ion batteries. When the cell system is exposed to extreme deformation, a simple cooling process can repair the fractured electrode-electrolyte interface, resulting in *in-situ* recovery of electrochemical performance compared to conventional flexible cells (Figure 7C). This process can be repeated even after multiple strong folding events, and this cooling-recovery strategy requires only external temperature stimulation without any side effects involving phase transitions. (Zhao et al., 2017).

Soorathep Kheawhom’s group also reported a transparent alkaline GPE film, which is polymerized by polyacrylic acid, potassium hydroxide and water as a quasi-solid electrolyteused in zinc-manganese dioxide batteries. The GPE film has high ionic conductivity, improveing the cycle life and performance of the battery. (Gaikwad et al., 2011; Lao-atiman et al., 2017).

The application of organic electrolytes in zinc ion batteries is still the least explored field in stable zinc anodes. Due to the higher thermodynamic stability of Zn to organic solvents, the application of organic solvents may reduce side reactions that may solve the lower Coulombic efficiency (CE) problem by simplifying electrochemistry at the Anode and reducing electrode passivation, thereby reducing dendrite problems.

Wang and colleagues reported that a highly stable and dendrite-free zinc anode was obtained by using triethyl phosphate (TEP) as a cosolvent in aqueous solution. In more than 3,000 h stable galvanized iron/zinc, CE value of 99.68%. The electrolyte and zinc ion battery of zinc anode and six iron acid potassium cyanide copper (KCuHCf) positive electrode showed a good compatibility. The full battery has longer cycle stability and higher rate performance. (Naveed et al., 2019).

However, compared with stream electrolytes, organic electrolytes are less safe, so stream electrolytes are the most widely studied. The flexible zinc ion battery also requires that the electrolyte can withstand the test of bending and folding, and has a certain self-healing ability.

Wong and colleagues developed a simple and economical method to construct a versatile hydrogel electrolyte using cotton as raw material, tetraethyl orthosilicate as crosslinking agent, and glycerin as antifreeze agent (Chen et al., 2021b). The resulting hydrogel electrolyte has high ionic conductivity, excellent mechanical properties (such as high tensile strength and elasticity), ultra-low freezing point, good self-healing ability, high adhesion and good heat resistance. It is worth noting that this water gel electrolyte under - 40°C can provide a record of 19.4 mS/cm high ionic conductivity. Secondly, the hydrogel electrolyte can significantly inhibit zinc dendrite growth and hydrogen evolution side reactions at -40–60°C (Figures 7D,E). Using this hydrogel electrolyte, a flexible quasi-solid Zn-MnO_2_ cell was assembled, which showed significant energy density in the -40–60°C range. The battery also shows excellent cycle durability, with high durability in a wide range of harsh conditions. In addition, it can be in >60% of compression deformation restored to its initial state, shows that it has good elasticity. Zhi’s team reported cathodes based on nanofibrillated cellulose (NFC)/polyacrylamide (PAM) hydrogels, electrodeposited Zinc nanoplate anodes, and carbon nanotube (CNT)/α-MnO_2_ sewable Zn-MnO_2_ cells (Wang et al., 2018a). The designed NFC/PAM hydrogel has high mechanical strength and great tensile properties. The prefabricated NFC bone network stabilizes the large pore as a channel for electrolyte diffusion. In addition, the influence of sewing on improving shear resistance of solid battery was analyzed. The sutured Zn-MnO_2_ battery retains 88.5% capacity after 120 stitches and can withstand a large shear force of 43 N.

In order to effectively solve the disadvantage of rapid water loss of hydrogel electrolyte, Liu and colleagues report a solid-state battery based on a self-standing gelatin-based hydrogel electrolyte (GHE) (Han et al., 2018b), whose high ionic conductivity, strong mechanical strength, and tight contact with the electrode enable reversible and stable circulation of Zn metal anodes and LiMn_2_O_4_ cathodes. Benefiting from superior stability to water, GHE’s flexibility and a carefully designed battery structure, the Zn/GHE/LiMn_2_O_4_ solid state full battery delivers a high specific capacity of 110.2 mA h/g and is resistant to cutting, flooding, bending, twisting and crimping. These characteristics of gelatin electrolyte proved beneficial to the battery components and battery performance. When heated, gelatine powder dissolved in AE, which promoted *in-situ* coating of GHE on the electrode. Using the fast cooling technology after cooling, gelatin solidifies into a solid layer of thin film, to provide mechanical strength to suppress the formation of zinc dendrite.

In a word, the research of flexible zinc ion battery electrolyte mainly focuses on how to alleviate the problem of zinc negative dendrite, hydrogen evolution and solve the problem of hydrogel electrolyte water loss. High performance electrolytes have great influence on the cycle stability, rate performance and energy storage capacity of batteries. (Liu et al., 2019b; Ma et al., 2019b). Therefore, it is of great significance to study electrolytes of flexible Zn-ion batteries.

## Method of Combining Active Materials With Flexible Substrates

In order to improve the mechanical flexibility of electrodes for wearable electronic devices so that the active material can better adhere to the flexible substrates, flexible electrodes are usually prepared by two ways: one method is to grow the active material directly on the flexible substrate, which is typically carbon cloth, polymer elastomers or textiles. Direct growth of active materials without using any binder, such as catalytic *in-situ* growth, electrodeposition (Liu et al., 2020; Yza et al., 2022), etc. Another method is to load active material with the help of instrument. This includes loading of electrically active material onto flexible substrates by screen printing with the help of adhesives, or loading the active material by inkjet printing, 3D printing, or laser etching with a pre-designed electrode pattern.

### *In situ* Growth Method

In the preparation of zinc ion batteries, the use of polymer binders and conductive additives results in high contact resistance, which reduces the specific capacity and diversity performance of the battery. An effective strategy to solve this problem is to directly grow the active material *in-situ* on flexible substrates or collectors as binder-free electrodes. (Wan et al., 2018). This strategy not only ensures fast charge transfer between the active material and the collector, but also enables a uniform distribution of the active material over the collector fluid. Therefore, the prepared flexible electrodes will provide better electrochemical performance, including high capacity, good rate performance and high capacity under deformation conditions.

**FIGURE 8:**
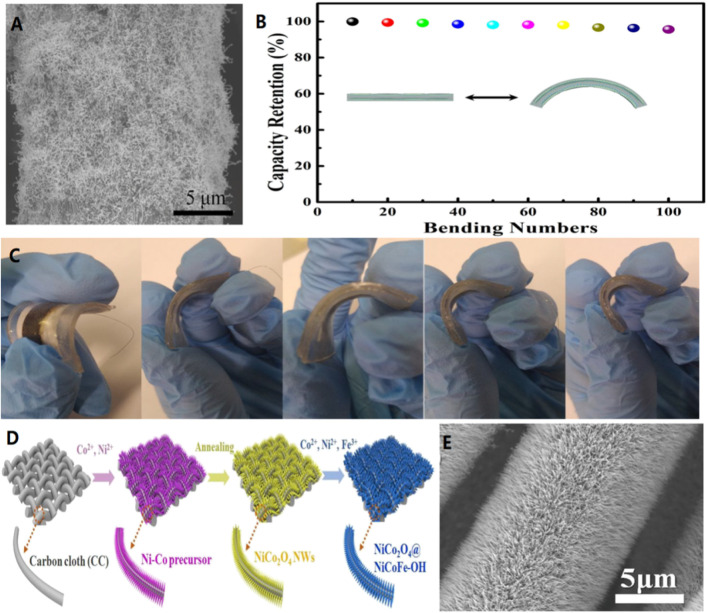
**(A)** SEM of MOF derived Ag nanowires/CC at Ar/NH_3_. **(B)** Normalized capacity of the quasi-solid-state Ag-Zn battery bent 135°for 100 cycles. **(C)** The photographs of bent quasi-solid-state Ag-Zn battery.Copyright 2018 American Chemical Society. **(D)** Scheme of the synthetic process of NiCo_2_O_4_@NiCoFe-OH. SEM images of NiCo_2_O_4_ NWs **(E)** grown on CC. Copyright 2020 Journal of Materials Chemistry A.

Yao’s team constructed a planar flexible quasi-solid aqueous rechargeable silver-zinc battery using silver nanowires made from a metal-organic framework (MOF) as an unbonded cathode on a carbon cloth. The results show that the ag-Zn battery has a significant energy density of 1.87 mWh/cm^2^ due to the abundant reaction sites and short electron and ion diffusion paths provided by the MOF Silver nanowires (Figure 8A). After bending 135° and cycling 100 times, its capacity remained above 93% (Figure 8B), which further demonstrates the superb mechanical properties of the prepared devices (Figure 8C). (Li et al., 2018c) In addition, Cai and colleagues prepared three-dimensional (3D) integrated binder-free dual-functional oxygen electrodes composed of NiCo_2_O_4_@NiCoFe-hydroxide (NiCo_2_O_4_@NiCoFe-OH) nanoarrays supported by carbon cloth (Figure 8D). The three-dimensional porous structure and good hydrophilicity of the nitrate-treated carbon cloth substrate facilitated the directional growth of NiCo_2_O_4_ nanocrystals. Therefore, the nickel-iron-hydroxide can randomly anchore and cover on the NiFe surface. Using this controllable and cost-effective *in-situ* synthesis strategy, the core/shell structure of NiCo_2_O_4_@NiCoFe-OH nanoarrays is tightly attached to the 3D interconnected carbon microfibers. (Figure 8E).The manufactured monolithic binderless oxygen electrode is freestanding and highly flexible/bendable, inheriting a high mechanical strength carbon skeleton. (Li et al., 2020c).

### Screen Printing Method

**FIGURE 9:**
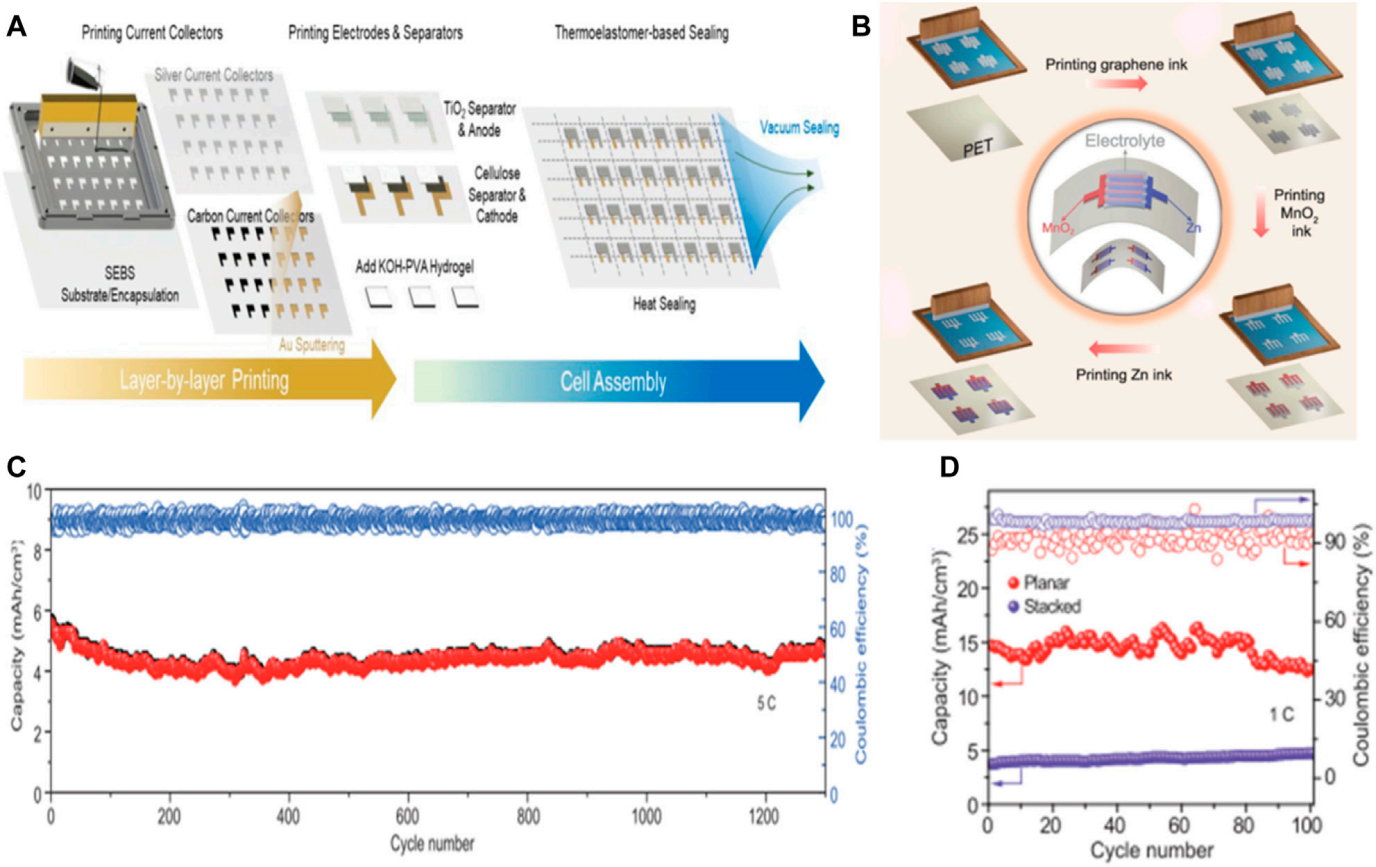
**(A)** The process of assembling the flexible, rechargeable, and high-capacity AGO-Zn battery is silkscreen printed layer by layer and vacuum sealed. Copyright 2019 ResearchGate. **(B)** Zn//MnO_2_ planar MBs was prepared by printing. Schematic diagram of printing zinc//MNO_2_MB by screen printing method: Blank PETsubstrate, printing graphene collector electrode, printing MnO_2_ cathode and Zn anode. Electrochemical performance of printed Zn//MnO_2_ planar MBs **(C)** cycling stabilities of printed Zn//MnO_2_ MBs with planar and sandwich-like stacked geometries, measured at a rate of 1 C; **(D)** long-term cycle stability of Zn//MnO_2_ planar MBs, with a high rate of 5C over 1,300 cycles. Copyright 2020 Natl Sci Rev.

Many flexible batteries rely on complex, low-production and high-cost manufacturing processes that hinder their transition from the lab to the marketplace, so printed high-performance batteries offer ideas for mass production of flexible batteries. Screen printing is considered to be a cost-effective, easy-to-operate and mass-productable method for the rapid construction of flexible zinc ion cells with precise control performance, flexibility and integration with printed microelectronics. (Guo et al., 2018). Screen printing allows active design control and can potentially combine deterministic and stochastic composites. In order to meet the need for flexibility and scalability while maintaining low cost and using low-cost thick film manufacturing techniques, flexible cell modules can be printed sheet by sheet or roll by roll using traditional and low-maintenance screen printing or scraper casting equipment, thus achieving low-cost mass production of flexible batteries (Figures 9A,B). (Kumar et al., 2016; Yin et al., 2021)

Wu’s team reports on a cost-effective and industrially applicable screen printing strategy for the rapid and scalable production of rechargeable zinc-manganese dioxide planar batteries with high performance, superior flexibility, scalable applicability and high safety. With zinc ink as anode (6.4 μm thick) and MnO_2_ ink as cathode (9.8 μm thick), high-quality graphene ink was used as metal-free collector in neutral electrolyte (2 M ZnSO_4_ and 0.5 m MnSO_4_). Planar unbaffled Zn//MnO_2_ MBs, tested in neutral aqueous solution, has a high capacity of 19.3 mA h/cm^3^ (corresponding to 393 mA h/g) at a current density of 7.5 mA/cm^3^, and a significant volume energy density of 17.3 mWh/cm^3^, superior to lithium film battery (≤10 mWh/cm^3^). In addition, the battery has stable cycling performance, with a capacity retention rate of 83.9% after 1,300 cycles under 5C, which is better than the previously reported stacked Zn//MnO_2_ battery (Figures 9C,D). (Wang et al., 2020)

Screen printing solves the requirement of high volume production of flexible electrodes at low cost, but faces another difficulty, which is to select or develop the ink formulation and choose the mesh size, as well as the thickness of each layer of ink film forming the anode and cathode, according to the requirements of the amount of active material, electrical properties, electrode stability and electrolyte permeability. (Tehrani et al., 2015). The selection of the mesh is based on the particle size of the mass of active material to be printed. A mesh with too large an aperture loses the significance of screen printing, while a mesh with too small aperture leads to uneven printing of the active material, which in turn affects the electrochemical properties. The right printing template will allow the active substance to be printed more evenly and firmly and to meet the required amount of active substance.

The rheology of the ink is controlled by a complex formulation of reactive materials, binders and specific solvents. The role of the binder is to bond the ink components and the collector fluid together and to influence whether the active substance will come off. Ink with different ratios of the same active material can also have a great impact on the battery performance. Somenath Mitra’s team reported the preparation of printable composite electrodes embedded with multi-walled carbon nanotubes (CNTs) for flexible Ni-Zn batteries. (Wang et al., 2018b). Carbon nanotubes function for electron transport pathways, resulting in better performance than physical mixing of active materials. However, excessive amounts of carbon nanotubes can lead to cracking of the active material. Therefore, it is extremely important to test a suitable ink formulation, which is an inevitable requirement for the preparation of cells with excellent electrochemical properties.

### Inkjet Printing Method

Inkjet printing, as an environmentally friendly and low-cost method for direct deposition of functional nanomaterials, has received widespread attention. Non-contact printing can be achieved on various substrates by programming the print pattern to be printed and then controlling the movement of the print nozzle. The modes of operation for inkjet printing are (i) drop-on-demand (DoD) printing, which delivers droplets caused by thermal bubbles or piezoelectric actuators, and (ii) continuous inkjet (CIJ) printing, which produces a continuous stream of ink through the nozzle by means of an electrostatic field or magnetic field. (Zhang et al., 2020e; Yan et al., 2020).

**FIGURE 10:**
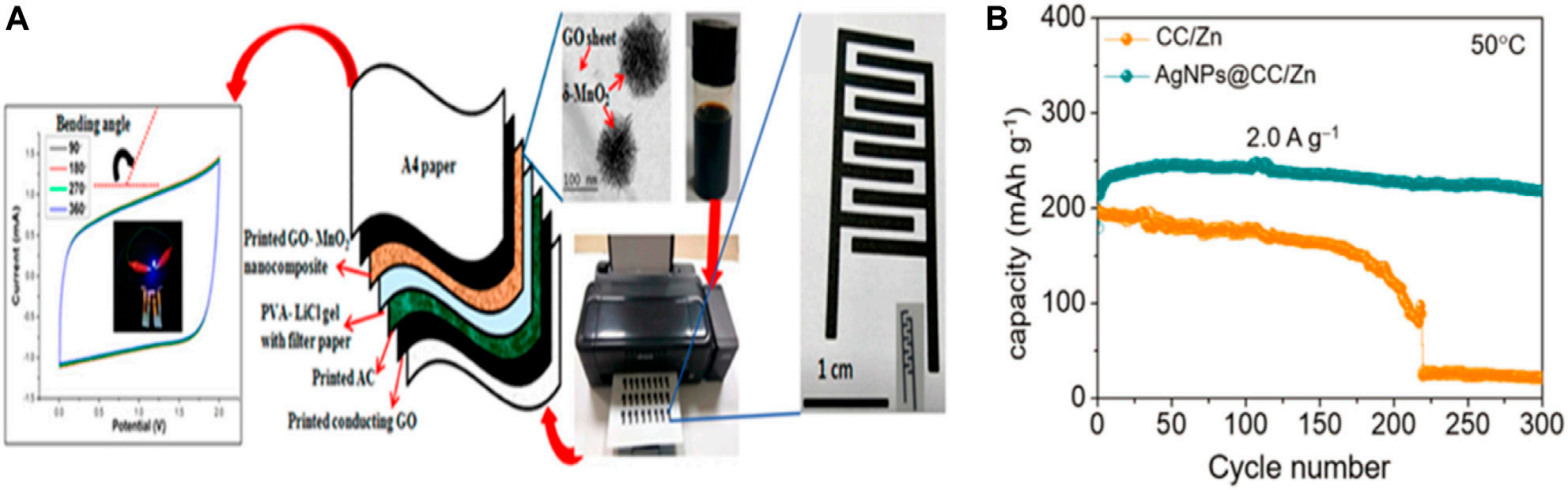
**(A)** Schematic of inkjet-printed MnO_2_/GO-based flexible supercapacitors on A4 paper substrates using an EPSON L130 printer. Left: CV curves of the flexible supercapacitors under various bending angles. Reproduced with permission from P. Copyright 2017 American Chemical Society. **(B)** Use the CC/zinc and AgNPs @ CC/zinc anode NVO lots zinc battery cycle stability under the condition of 50°C.Copyright 2021 Advanced Functional Materials.

Ink-jet printing provides the advantage of flexible and wearable electronics manufacturing, such as design of high-throughput, large-scale, good performance, biocompatibility, and on the infinite basal precision deposition. In addition, due to droplet deposition and programmable pattern design, inkjet printing methods greatly reduce the waste of expensive ink materials (Figure 10A).

Chen and his colleagues proposed a heterogeneous metal seed-mediated strategy. The basic idea is to print silver nanoparticles on a three-dimensional conductive backbone by inkjet printing and use them as heterogeneous metal seeds to induce uniform nucleation of zinc and avoid dendrite growth during the initial electroplating stage.At the same time, the reaction of Ag with Zn produces a zinc-friendly AgZn_3_ alloy as a Zn resource to compensate for the irreversible loss of active Zn during the recycling process. In the first 15 cycles, the capacity of the AgNPs@CC/Zn anode cell increased from 182 mA/g to 237 mA/g and maintained a reversible capacity of 218 mA/g, corresponding to a capacity retention rate of 92% (Figure 10B). (Chen et al., 2021a)

However, at present, there are still many challenges that the technology is relatively bad. These challenges include preventing nozzle clogging, inhibiting coffee ring effect, and enhancing ink dispersion,etc. (Yan et al., 2020)

### Other Methods

**FIGURE 11:**
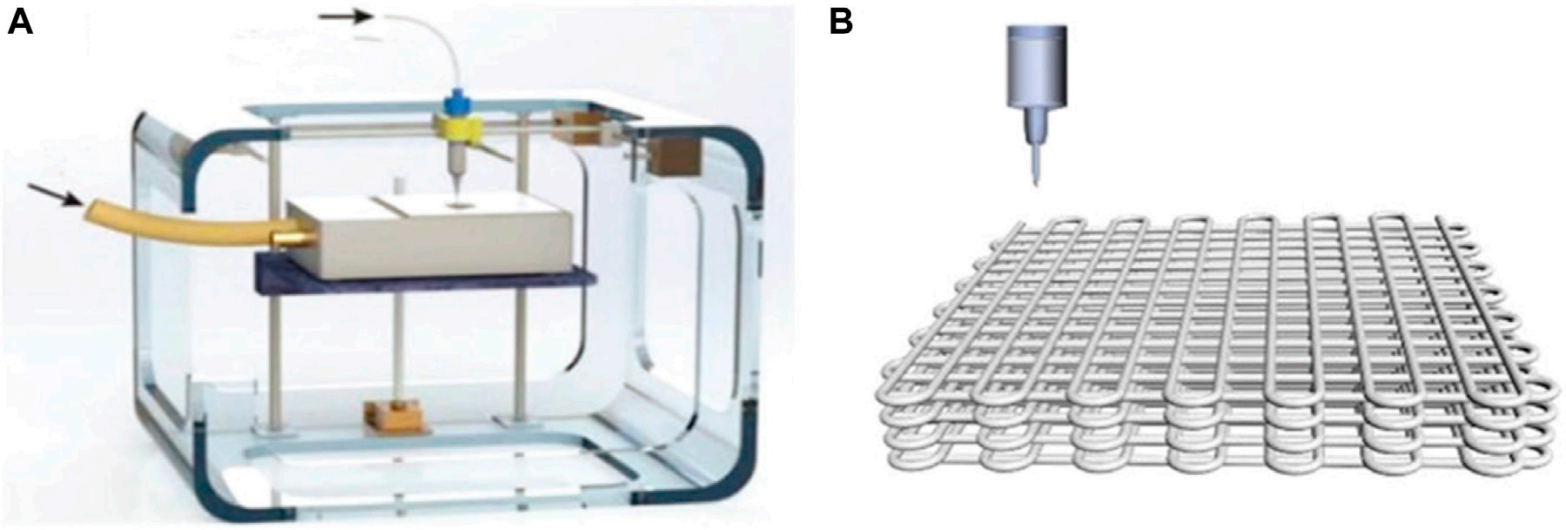
**(A)** Schematic of the 3D “drop-on-demand” ink jet printer.Copyright 2019, Elsevier. **(B)** Schematic illustration of 3DP-Zn-E. Copyright 2019, Advanced Functional Materials.

Those climb from claiming 3D printing (3DP) innovation Concerning illustration An revolutionary manufacturing system has created a great attraction for the fabrication of functional electrodes in the field of energy storage and conversion. The printing process usually combines digital programming and manufacturing procedures to rationally design a 3D functional structural model of the object, followed by the use of special software to obtain sliced layers of the model, which are printed using digital technology material printers, i.e. by adding each two-dimensional (2D) layer to the previous one, enabling the construction of a 3D structure, thus enabling precise control of the spatial geometry and architecture of the device from the macro to the nano scale (Figure 11A). (Pang et al., 2019) Yang’s team has demonstrated a 3D printing system for those development of practical electrodes for zinc-air batteries. The printed anode comprises for large portions minor zinc balls. to enhance the utilization of zinc. The self-supporting air cathode made by 3D printing has a hierarchical porous structure with high specific surface area, which allows the electrode to have high electrocatalytic activity and fast reaction diffusion channels (Figure 11B). Thanks to these advantages, the assembled zinc-air battery achieves a high releasing capacity of 670 mA h/g at a current density of 5 mA/cm^2^ and a long period cycle stability of at least 350 cycles. (Zhang et al., 2020f).

Laser engraving processing is based on the use of CNC technology and laser as the processing medium. The physical denaturation of the electrode material by instantaneous melting and vaporization under laser engraving irradiation enables laser engraving to achieve the processing purpose, which in turn completes the preparation of flexible electrodes.Wang’s team used a high-precision, simple and low-cost laser engraving technique to prepare finger-shaped cathodes and anodes for ZIMB. Suspended finger-like multi-walled carbon nanotube MnO_2_ (MWCNTs-MnO_2_) cathodes were prepared by laser engraving machine (JL-K3020) and MWCNTs-Zn anodes based on zinc nanosheets were prepared by laser etching with further acknowledge the full adaptability from claiming ZIMB. The flexible conductive substrate prepared by laser engraving allows ZIMB to exhibit excellent flexibility, resulting in high reliability and stability of ZIMB. The concept proves that the capacity maintenance of ZIMB is up to 96.5% of the beginning capacity at 120° bending angle, and even after the 5th self-healing cycle, the capacity retention is still up to 90.2%. This worth of effort gives new plans for outlining another era of high-efficiency, high-reliability and high-stability micro-energy storage devices. (Du et al., 2020).

## Summary and Outlook

In the era of electronic information technology, electronic devices are developing towards miniaturization and flexibility. Those ever-growing enthusiasm from both academic Group What’s more shopper showcase will be activating those fast advancement of new helter skelter execution adaptable vitality stockpiling gadgets. In the research of flexible power supply, flexible zinc ion batteries have been noticed by researchers and have achieved some promising results. In this paper, the research progress of flexible zinc batteries in recent years is reviewed from the following aspects: the differences between different anode, cathode materials and electrolytes in flexible zinc batteries; the method of loading active materials on flexible electrodes are discussed and compared. Although many progresses have been made in this area, it is still highly desirable to solve the following challenges and problems in time to promote the application of flexible zinc batteries.

First of all, the performance of the active materials has a critical impact on the cycling performance and flexibility of the battery. The research of zinc anodes mainly focuses on the material of metal zinc anodes. The primary goal is to reduce the generation of zinc dendrites during long-term cycling, so as to improve the lifetime and electrochemical performance of the battery. Most studies have used zinc foil directly, and some studies have cast zinc and zinc oxide particles on flexible substrates or grow zinc *in situ* instead of zinc foil in order to obtain better mechanical properties and stability. The research of cathodes is mainly to try to obtain materials with high capacity, high cycling performance, low cost and good mechanical properties, including ion embedding and oxygen vacancy generation for some cathode materials to improve the battery performance. Moreover, the research on the electrolyte of flexible zinc ion battery mainly focuses on the gel electrolyte. It consists of polymer body and liquid electrolyte, which has relatively excellent flexibility performance and can basically meet the needs of flexible zinc ion battery, and also has relatively good ionic conductivity to improve the multiplication performance of flexible zinc ion battery, as well as can make good interfacial contact with electrode.

Secondly, the main technologies for loading active materials onto flexible electrodes include *in-situ* growth method, screen printing, and inkjet printing and so on. Among them, the *in-situ* growth of active materials does not require binder, which ensures rapid charge transfer between active materials and collectors, makes the active material evenly distributed on the collector, so that the prepared flexible electrodes have better electrochemical performance. Screen printing is a sheet-by-sheet or roll-by-roll printing method for flexible cell, which is considered to be a cost-effective, easy-to-handle and mass-producible process. Inkjet printing can programmed according to the pattern to be printed, enabling non-contact printing on a variety of substrates, resulting in low-cost, high-precision, high-efficiency electrode printing, which can be used for artificial intelligence equipment, special-shaped battery and private customization.

With the innovation of technology, flexible zinc ion battery has a wide application prospect in flexible electronic devices due to its excellent performance. The development of wearable/implantable electronic devices such as flexible display devices, health monitors and electronic sensors has attracted more and more attention from academia and industry. One of the biggest challenges in the development of flexible electronic devices is to develop flexible, light, thin and safe portable energy storage devices. Flexible water zinc ion batteries are safe, can meet safety performance, and can be designed to fit into clothing and other wearable devices. The batteries must better fit the shape of wearable devices to avoid being bulky. Therefore, the development prospect of flexible zinc ion battery is broad.

Despite the advantages of flexible zinc batteries, there are still many problems for researchers to continue to study: (1) compared with the high theoretical capacity of the zinc metal anode, the cathode active material with higher capacity need to be explored; (2) the stability of the zinc negative electrode at high current and high capacity needs to improved; (3) in flexible batteries, with movements such as bending, the loaded active material usually cracks or flakes off, leading to a rapid decline in electrochemical performance. Therefore, it is necessary to design the ideal electrode material structure or improve the process method of loaded active material, and then further improve the mechanical properties of the electrode; (4) to further increase the actual capacity of flexible zinc batteries while improving the mechanical properties of electrodes. How to encourage the capacity of zinc ion batteries by increasing the amount of loaded active substance, which in turn has wider practicality.
